# Data regarding the effects of thrombin and dabigatran-inhibited thrombin on protease-activated receptor 1 and activation of human atrial fibroblasts

**DOI:** 10.1016/j.dib.2018.05.124

**Published:** 2018-05-25

**Authors:** Paola Altieri, Maria Bertolotto, Patrizia Fabbi, Elena Sportelli, Manrico Balbi, Francesco Santini, Claudio Brunelli, Marco Canepa, Fabrizio Montecucco, Pietro Ameri

**Affiliations:** a Laboratory of Cardiovascular Biology, Department of Internal Medicine, University of Genova, Genova, Italy; b Department of Internal Medicine, University of Genova and First Clinic of Internal Medicine, IRCCS Ospedale Policlinico San Martino, Genova, Italy; c Department of Diagnostic and Surgical Sciences, University of Genova and Cardiovascular Surgery Unit, IRCCS Ospedale Policlinico San Martino, Genova, Italy; d Cardiovascular Disease Unit, IRCCS Ospedale Policlinico San Martino, Genova, Italy; e Centre of Excellence for Biomedical Research (CEBR), University of Genova, Genova, Italy

## Abstract

The data presented here are related to the research paper entitled “Thrombin induces protease-activated receptor 1 signaling and activation of human atrial fibroblasts and dabigatran prevents these effects” (Altieri et al., 2018) [1]. Data show that silencing of protease-activated receptor 1 (PAR1) prevents the activation of Fib isolated from atrial appendages of patients without atrial fibrillation (AF), as assessed by immunofluorescence for α-smooth muscle actin (αSMA) and Picro-Sirius red staining. Moreover, it is reported that primary atrial Fib obtained from two subjects with permanent AF express PAR1 and PAR2 and display enhanced αSMA immunoreactivity and collagen synthesis in response to thrombin, but not to dabigatran-bound thrombin, alike Fib from non-fibrillating atria.

**Specifications Table**

**Table:** 

Subject area	*Health science*
More specific subject area	Experimental cardiology
	Atrial fibrillation; cardiac fibroblasts
Type of data	*Text file, figures*
How data was acquired	*Microscope (Leica DM2000 fluorescence microscope and Olympus BX50 light microscope, coupled to Leica Application Suite software)*
Data format	*Analyzed*
Experimental factors	*Primary human atrial fibroblasts untreated or exposed to thrombin, dabigatran-bound thrombin or dabigatran alone*
Experimental features	*Silencing of PAR1 attained by small interfering RNA-based knockdown*
	*Protein expression analyzed by immunofluorescence and western blot, collagen synthesis by Picro-Sirius red staining, and PAR1 expression by RT-PCR*
Data source location	*Laboratory of Cardiovascular Biology, Department of Internal Medicine, University of Genova, Genova, Italy*
Data accessibility	*Data are available within the article*
Related research article	*“Thrombin induces protease-activated receptor 1 signaling and activation of human atrial fibroblasts and dabigatran prevents these effects” by Altieri P, Bertolotto M, Fabbi P, Sportelli E, Balbi M, Santini F, Brunelli C, Canepa M, Montecucco F, Ameri P.*

**Value of the data**

• Data expand on the knowledge of thrombin actions on human cells.
• Data may provide the background for further studies about PAR1-mediated effects of thrombin on human atrial fibroblasts in both the absence and presence of AF.
• Data may serve as reference for silencing of target genes in primary human atrial fibroblasts.

## 1 Data

**Fig. 1:**
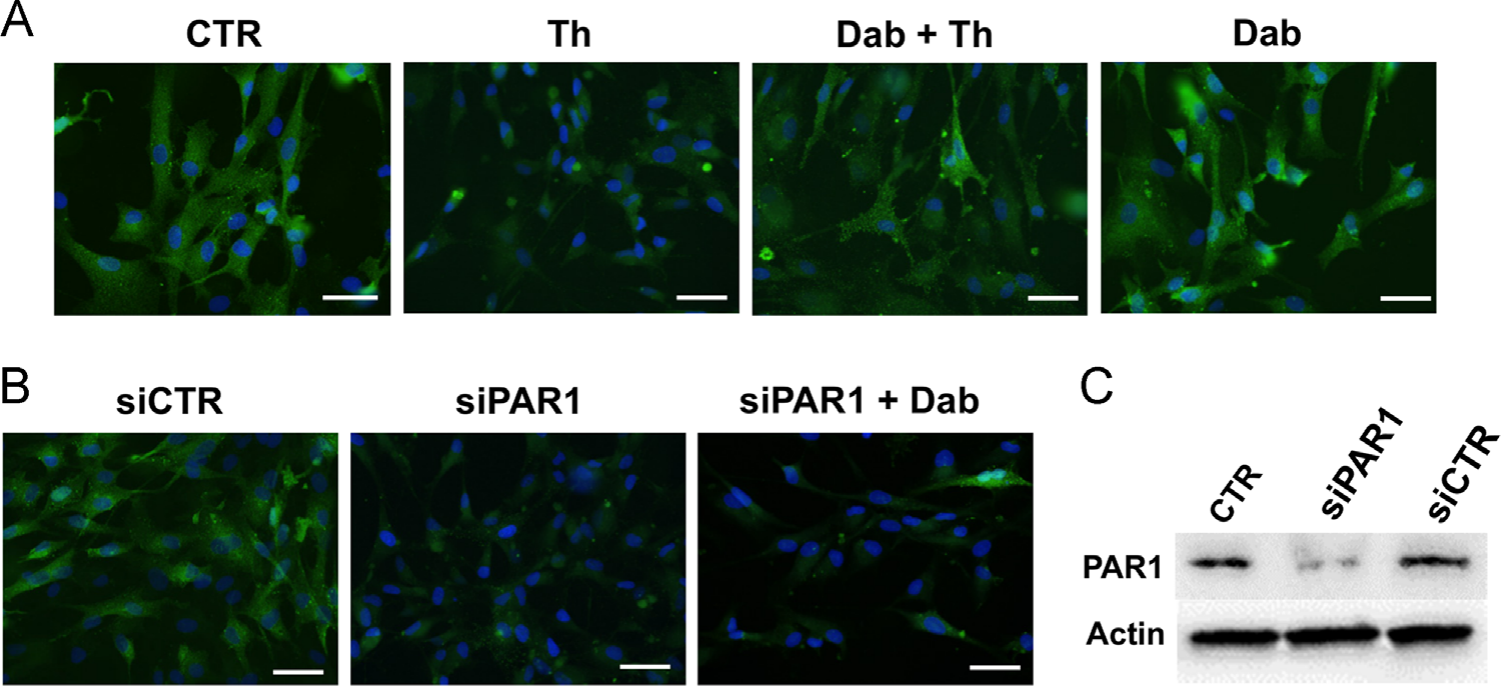
**(A)** Representative pictures of primary human atrial Fib from patients without AF, immunostained for PAR1 after no treatment or exposure to thrombin (Th), dabigatran followed by thrombin (Dab + Th), or dabigatran alone (Dab). (**B**) Representative pictures of immunofluorescence for PAR1 in primary Fib from non-fibrillating atria, transfected with negative control siRNA (siCTR) or siRNA targeting human PAR1 (siPAR1), with or without subsequent incubation with dabigatran (Dab). Magnification is 200×, bars indicate 50 μm. (**C**) Western blot for PAR1, confirming the knockdown of PAR1 protein after transfection with siPAR1, but not siCTR.

**Fig. 2:**
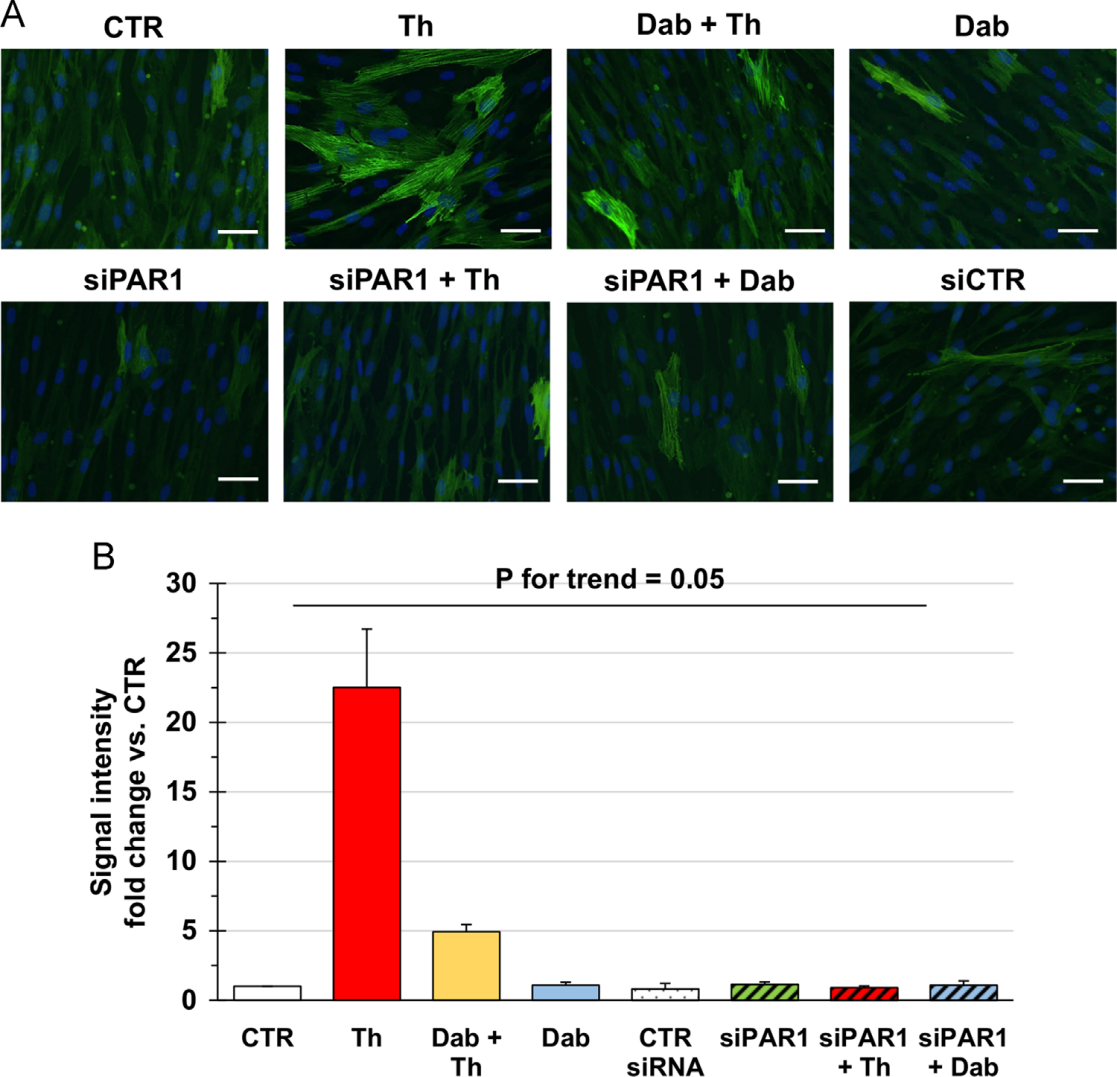
Representative images (**A**) and quantification (**B**) of immunofluorescence for αSMA in primary human atrial Fib left untreated (CTR), incubated with thrombin (Th) and/or dabigatran (Dab), or transfected with a control (siCTR) or a PAR1-targeting (siPAR1) siRNA with or without subsequent exposure to Th or Dab as indicated. Fib were obtained from subjects with no history of AF. Magnification 200×, bars 50 μm. Data in B are from three independent replicates and were compared by the Kruskal-Wallis test.

**Fig. 3:**
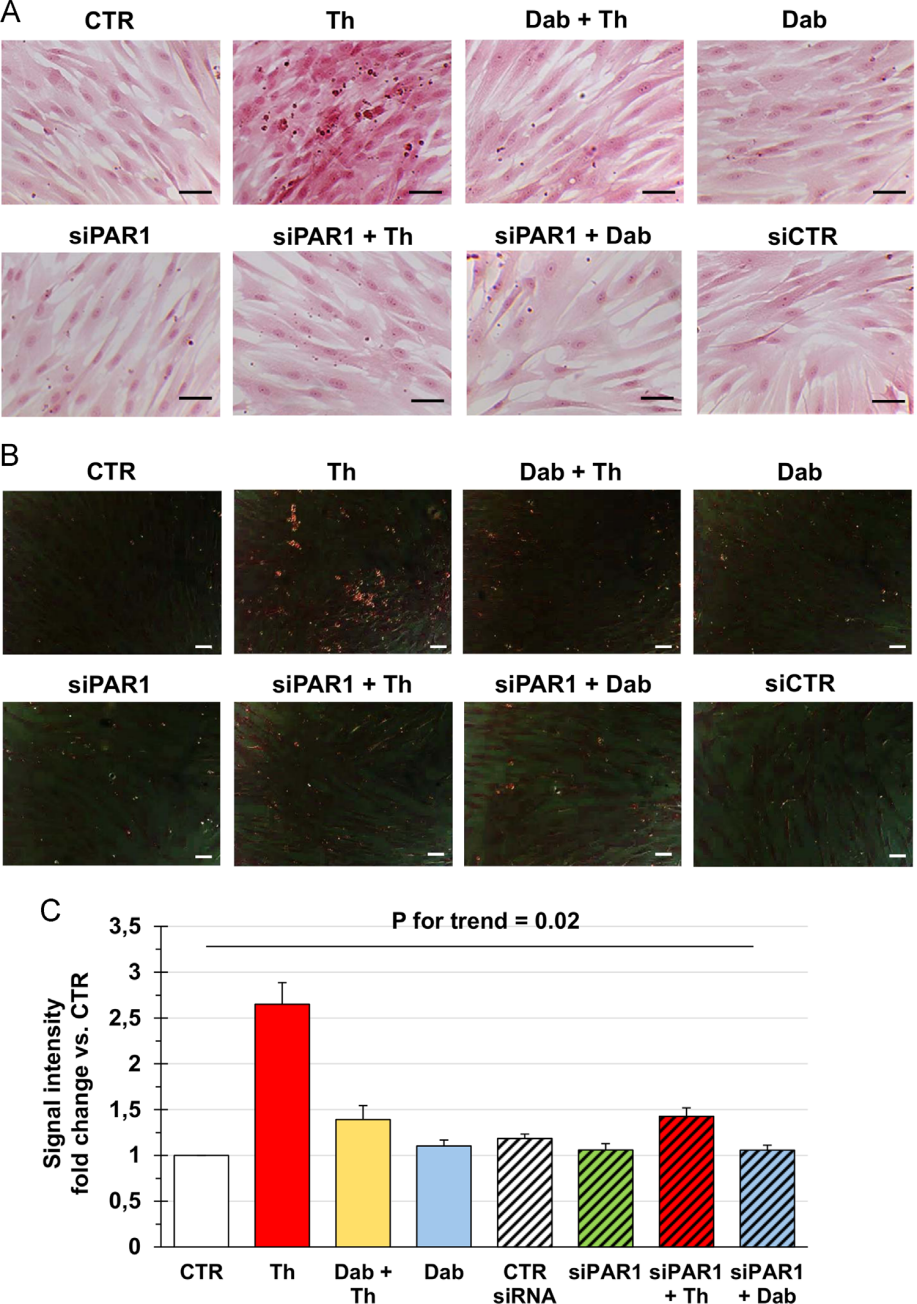
Primary atrial Fib from subjects without AF were left untreated (CTR), exposed to thrombin (Th) and/or dabigatran (Dab), or transfected with a control (siCTR) or a PAR1-targeting (siPAR1) siRNA with or without subsequent incubation with Th or Dab. At the end of treatments, cells were stained with Picro-Sirius red. Representative images of the staining in conventional and polarized light microscopy are given in (**A**) and (**B**), respectively, while quantification of the red signal in conventional light microscopy is presented in (**C**). Magnification is 200× and bars correspond to 50 μm. Data in the graph are from three independent replicates and were compared by using the Kruskal-Wallis test.

Data confirm that thrombin cleaves PAR1 of primary human atrial Fib, this effect being antagonized by the direct thrombin inhibitor dabigatran (Fig. 1A), and show that small interfering RNA (siRNA)-based knockdown effectively reduces the amount of PAR1 protein, regardless of the presence of dabigatran (Fig. 1B–C). While thrombin, but not dabigatran-inhibited thrombin, substantially increased the expression of αSMA and the synthesis of collagen in control conditions, these effects were not observed when PAR1-silenced cells were incubated with thrombin (Fig. 2, Fig. 3).

To integrate the data presented above, which were obtained with cells harvested from the atrial appendages of patients without AF, atrial Fib were also collected from 2 subjects who instead suffered from permanent AF. Fib from fibrillating atria expressed PAR1 and PAR2 (1/2^DCt for PAR1 = 0.008 in both cases, 1/2^DCt for PAR2 = 0.001–0.004); mRNA for PAR3 was very low (1/2^DCt = 0.00003), and that for PAR4 not detectable.

**Fig. 4:**
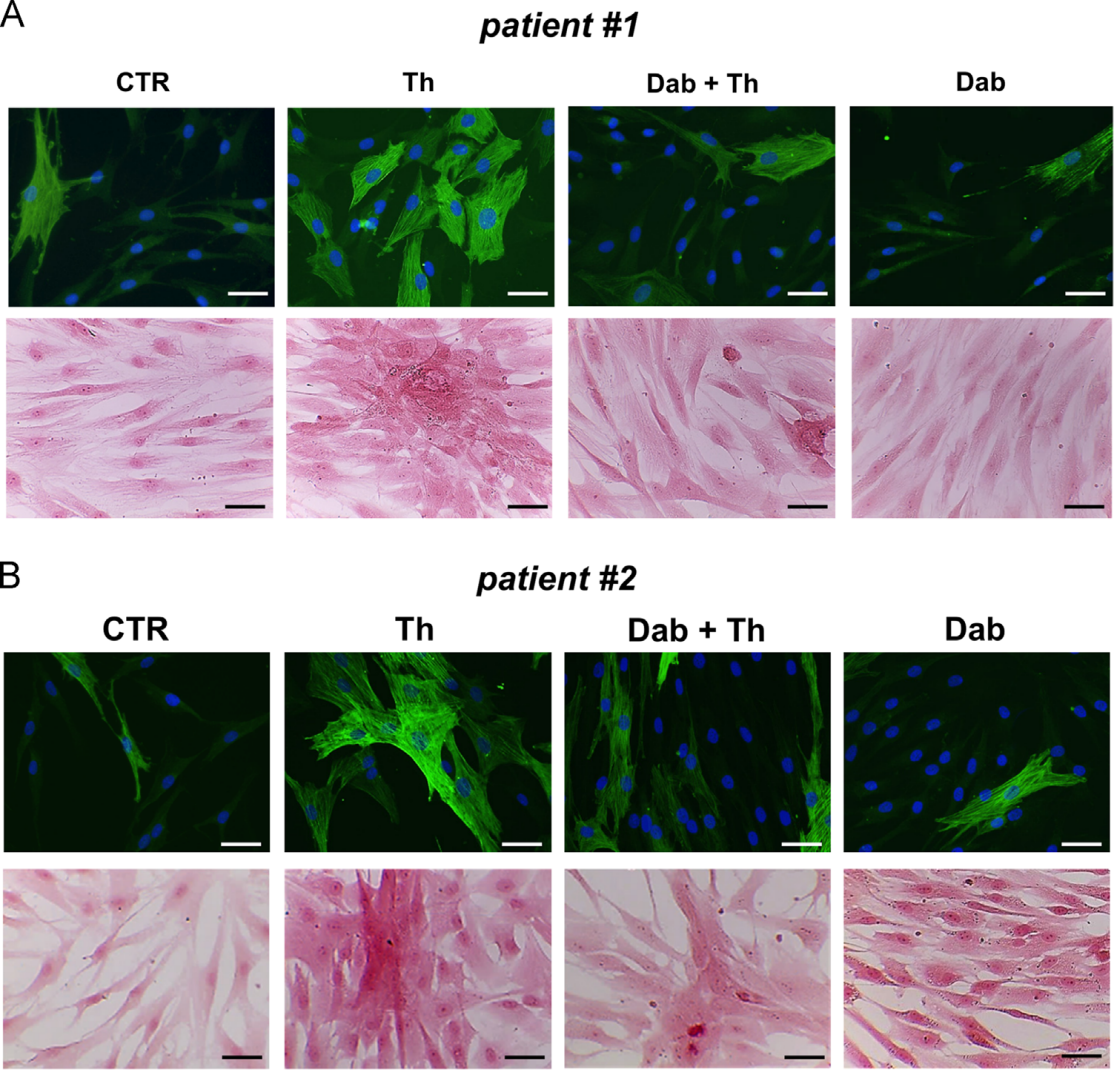
Representative images of immunofluorescence for αSMA (upper line) and Picro-Sirius red staining (lower line) of primary human atrial Fib isolated from the 2 patients with AF included in this study, after no treatment (CTR) or incubation with thrombin (Th) and/or dabigatran (Dab).

As compared with control, αSMA levels and collagen production significantly increased upon incubation with thrombin (Fig. 4). Like for Fib from non-fibrillating atria [1], dabigatran counteracted the accumulation of αSMA and collagen stimulated by thrombin (Fig. 4).

## 2 Experimental design, materials, and methods

### 2.1 Materials and reagents

Unless otherwise specified, materials and reagents were purchased from Sigma-Aldrich (Munich, Germany).

### 2.2 Cells and experimental design

Primary Fib were isolated from patients who underwent elective cardiac surgery: 8 without AF [1] and 2 with permanent AF, a 80 year old man with diabetes mellitus and a 78 year old woman with hypertension and dyslipidemia, both affected by valvular disease. Briefly, right atrial appendages were collected when the venous cannula was placed for extracorporeal circulation, fragmented and enzymatically digested for 45 min at 37 °C with 1 mg/mL collagenase II (Worthington Biochemical Corporation, Lakewood, NJ, USA) in Ca^2+^- and Mg^2+^-free PBS added with 0.2% BSA. At the end of the incubation, collagenase was neutralized by adding DMEM/F12 with 10% FBS and the resulting solution was filtered through a 70 μm nylon filter (BD Biosciences, Milano, Italy) and centrifuged for 10 min at 1400 rpm. The pellet was re-suspended in DMEM/F12 with 10% FBS and placed into a Petri dish. After 3 h, mostly Fib adhered to the bottom of the dish and non-adherent non-Fib cells were removed. Fib were then grown in Endothelial Growth Medium-2 (EGM-2; Lonza, Walkersville, MD, USA) to limit the tendency to spontaneous activation in response to culturing [2].

Cells were passaged at 70% confluence and used for experiments from passage 2 to passage 5. After washing twice with Endothelial Basal Medium-2 (EBM; Lonza), atrial Fib were treated with 1 nM thrombin (human α-thrombin at 3117 NiH U/mL specific activity; Enzyme Research Laboratories, South Bend, IN, USA), 500 nM dabigatran (Boehringer Ingelheim International GmbH, Biberach, Germany) for 30 min and then 1 nM thrombin, or 500 nM dabigatran alone. The duration of the incubation with thrombin was 15 min to evaluate the amount of total PAR1 and 48 h to assess αSMA and collagen deposition. All treatments were performed in EBM-2.

The study was approved by the local Ethics Committee and patients gave written informed consent to participate.

### 2.3 PAR1 silencing

Fib were seeded in 8-well chamber slides at a number of 5000 cells/well in antibiotic-free EGM-2, allowed to adhere overnight, and then transfected with Silencer Select Validated siRNA targeting human PAR1 (Ambion, Austin, TX, USA) or Silencer Negative Control #1 siRNA (ThermoFisher Scientific, Waltham, MA, USA) at a final concentration of 5 pMol, using Lipofectamine RNAiMAX (Invitrogen, ThermoFisher Scientific) as transfection reagent. After 24 h, the siRNA-lipofectamine solution was replaced with EGM-2. After another 24 h, cells were washed with PBS and exposed to thrombin, dabigatran for 30 min and then thrombin, or dabigatran alone in EBM-2. Treatment with thrombin lasted 15 min for assessment of total PAR1 levels and 72 h for evaluation of αSMA and collagen expression.

### 2.4 Analysis of PAR1 expression

RT-PCR for *F2R* (encoding PAR1) was carried out with Taqman Gene Expression Assay MTO from Life Technologies after extracting total RNA by means of the Quick-RNA MiniPrep kit (Zymo Research, Irvine, CA, USA) and reverse-transcribing to cDNA with iScript Reverse Trascription Supermix RT-qPCR (Bio-Rad). Samples were amplified by using an EagleTaq Universal master mix (Roche Diagnostics GmbH, Mannheim, Germany) on a CFX96 Touch RT-PCR Detection System (Bio-Rad). The expression of the genes of interest was normalized against that of *RPLP0* and analyzed with the comparative DDCt method. Assays ID were Hs00169258 and Hs99999902 for *F2R* and *RPLP0*, respectively.

Levels of PAR1 protein were investigated by immunofluorescence of Fib grown on chamber slides to 70% confluence. Cells were incubated for 1 h with a mouse monoclonal primary antibody against Arg27-Thr102 and Ser375-Thr425 of human PAR1 (clone 731115, R&D Systems, Minneapolis, MN, USA), then for 30 min with an Alexa Fluor® 488-conjugated secondary antibody (ThermoFisher Scientific). Nuclei were stained with DAPI (ThermoFisher Scientific).

PAR1 protein expression was also studied by Western blotting with overnight incubation with the mouse monoclonal antibody against Arg27-Thr102 and Ser375-Thr425 of human PAR1, followed by incubation with HRP-conjugated anti-mouse secondary antibody (Santa Cruz Biotechnology, Dallas, TX, USA). Protein bands were visualized with the Clarity Western ECL Substrate (Bio-Rad, Segrate, Milano, Italy).

### 2.5 Analysis of fibroblast activation and collagen secretion

Fib were cultured in 8-well chamber slides, treated for 48 or 72 h as described in 2.2, 2.3, and fixed in − 20 °C methanol for 1 min.

Expression of the marker of Fib activation, αSMA, was assessed by immunofluorescence with a mouse monoclonal anti-human αSMA antibody (clone 1A4, Dako, Denmark).

Collagen production was evaluated by Picro-Sirius red staining. Fixed cells were incubated for 1 h with a staining solution made of Sirius red (0.1%, Sigma) dissolved in a saturated aqueous solution of picric acid (1.3% in water, Sigma), washed three times with 0.1% acetic acid, and dehydrated with ethanol followed by xylene.

To quantify αSMA and collagen levels, random images were taken from at least 5 independent fields for each condition by using a Leica DM2000 fluorescence microscope (Leica Microsystems GmbH, Wetzlar, Germany) and a Olympus BX50 light microscope (Olympus, Tokyo, Japan), respectively, coupled to a CCD high resolution cooled camera and the Leica Application Suite software (Leica Microsystems). Signal intensity was then measured with the ImageJ analysis software (National Institutes of Health, Bethesda, MD, USA) [3].

### 2.6 Statistical analysis

Data are presented as mean ± SEM of fold change vs. control and were compared by the Kruskal-Wallis test with GraphPad Prism Version 6.0a (GraphPad Software). A *P* value <0.05 was considered statistically significant.
