# Supplementary material for: Data regarding the effects of thrombin and dabigatran-inhibited thrombin on protease-activated receptor 1 and activation of human atrial fibroblasts
Source: Data Brief. 2018 May 25;19:925–31. doi: 10.1016/j.dib.2018.05.124 (PMC5997940; doi:10.1016/j.dib.2018.05.124)
Supplement: Supplementary file 1 — Transparency document [file mmc1.docx]

Università degli Studi di Genova

DIPARTIMENTO DI MEDICINA INTERNA E SPECIALITÀ MEDICHE

16132 Genova – Viale Benedetto XV, 6 – Tel.+39 010 353.71 - +39 010 353.7900-7901 – Telefax +39 010 353.8638

**E-mail: dirdimi@unige.it – Partita I.V.A. 00754150100**

**Di.M.I.**

May 16^th^, 2018

**Ref: DIB-D-18-01217**

**Conflicts of interest concerning the article entitled “Data regarding the effects of thrombin and dabigatran-inhibited thrombin on protease-activated receptor 1 and activation of human atrial fibroblasts”.**

Dr. Ameri received consultant and speaker fees from Boehringer Ingelheim, Bayer and Daiichi Sankyo, and an investigator-initiated grant from Boehringer Ingelheim. The other authors have no conflicts of interest to disclose.

Best regards,

Pietro Ameri, MD, PhD

**Reviewer 1**

**All comments have been addressed, acceptance of this excellent mansucript is recommended.**

We thank the Reviewer for the valuable hints given and for appreciating the final manuscript.

**Reviewer 2**

**The Authors addressed many comments and the manuscript has improved, though there are a few issues remaining that are outlined below:**

**1. The Authors used Picro-Sirius red to assess collagen content; however, it has to be analysed appropriately (as picro-sirius red staining binds to both collagenous and non-collagenous fractions) and carefully (it is not clear how exactly the analysis was done apart from imaging the cells for “red” staining), although the Authors show corresponding images in polarized light in some (but not all) figures (e.g., suppl. Fig.3). Please use appropriate analysis of all picro-sirius red images (i.e., colorimetric assay and/or polarized light microscopy of the same images).**

We apologize for not being clear about the way Picro-Sirius red staining was quantified: we assessed the red signal intensity in conventional light microscopy images. This is now stated in the main manuscript, as well as in the Data in Brief file.

Manuscript, page 5, Methods, section 2.3: “*Collagen synthesis was also analyzed by Picro-Sirius red staining of Fib cultured in 8-well chamber slides; red signal intensity was quantified in images taken with a conventional light microscope.*”

Supplementary Material, section 2.4, page 4, lines 10-12 from top: “*The amount of collagen was measured as red signal intensity by analyzing images taken with an Olympus BX50 optical microscope (Olympus, Tokyo, Japan) with the ImageJ software.*”

Data in Brief file, section 2.5 of Experimental Design, Materials and Methods: “*To quantify αSMA and collagen levels, random images were taken from at least 5 independent fields for each condition by using a Leica DM2000 fluorescence microscope (Leica Microsystems GmbH, Wetzlar, Germany) and a Olympus BX50 light microscope (Olympus, Tokyo, Japan), respectively, coupled to a CCD high resolution cooled camera and the Leica Application Suite software (Leica Microsystems). Signal intensity was then measured with the ImageJ analysis software (National Institutes of Health, Bethesda, MD, USA) [3]*.”

Data in Brief file, legend to Fig. 3: “[…].*At the end of treatments, cells were stained with Picro-Sirius red. Representative images of the staining in conventional and polarized light microscopy are given in (A) and (B), respectively, while quantification of the red signal in conventional light microscopy is presented in (C)*.”

Furthermore, a graph with the quantification of Picro-Sirius red staining has been added to Supplementary Fig. 3, with the relevant legend: “*Quantification of Picro-Sirius red signal intensity in conventional light microscopy images from 3 independent experiments is presented in the graph. Data were compared by one-way ANOVA followed by post-hoc Tukey’s multiple comparisons test (overall P for trend value <0.0001, *** indicates P <0.001 in post-hoc Tukey’s test).*”

**2. In Fig. 1. B. “Representative pictures of primary human atrial Fib immunostained for PAR1” (Data in brief), the siRNA-mediated knockdown should be checked by a conventional method qRT-PCR or at least by PCR, and by Western blotting; the use of semi-quantitative immunofluorescence method is insufficient in such type of experiments.**

The Western blot for PAR1 has been added.

**3. Figures 3 and 4 (Data in brief) are exactly the same. Please provide the correct figures corresponding to the figure legends.**

We apologize for the mistake: the same figure (Fig. 3) was uploaded twice to the IJC website. Both Fig. 3 and Fig. 4 are now provided.

**In the same section, which patient group is shown in Figure 3, with or without atrial fibrillation? It is not stated in the figure legend.**

The Fig. presents data obtained with fibroblasts from patients without atrial fibrillation. The information, which was given in the Data section, is now also included in the legend: “*Primary atrial Fib from subjects without AF were left untreated (CTR), exposed to thrombin (Th) and/or dabigatran (Dab), or transfected with a control (siCTR) or a PAR1-targeting (siPAR1) siRNA with or without subsequent incubation with Th or Dab.*”

**4. In some figure the Authors use 1-way ANOVA for 3-4 samples (e.g., Suppl. Fig. 2); the normality test cannot be performed on such small sample size, therefore, such data should be treated and analysed by non-parametric statistical tests. This has been pointed out in previous review process, yet the Authors failed to carefully revise statistical analysis. Please check every figure and re-analyse appropriately where needed.**

Prompted by the Reviewer’s comment, we went over all the dataset and the statistical analyses. We followed the criterion stated in the Statistical Analysis section, i.e. absolute values were compared by one-way ANOVA, while the non-parametric Kruskal-Wallis test was used when data were fold change vs. control.

Manuscript, page 6, Methods, Section 2.10: “*Data are presented as mean ± SEM of absolute values or fold change vs. control and were compared by one-way ANOVA followed by post-hoc Tukey’s multiple comparisons test or Kruskal-Wallis test followed by post-hoc Dunn’s multiple comparisons test, respectively, by using GraphPad Prism Version 6.0a (GraphPad Software). Statistical significance was set at P < 0.05*.”

In fact, data in Fig. 4C, Supplementary Fig. 4B and Fig. 2 and 3 of the Data in Brief material have been re-evaluated by Kruskal-Wallis test, instead of one-way ANOVA as in the previous version of the manuscript. We thank the Reviewer for allowing us to note that the statistical test used for these data was inconsistent with the criteria presented in the Statistical Analysis section.

By contrast, we respectfully disagree about using a non-parametrical test just because of the small size of the samples. ANOVA is commonly employed to compare cell culture data in spite of the number of observations for each condition typically being small: this is the standard for major journals, including the International Journal of Cardiology.

**Minor comments to be addressed:**

**1. Line 200, ”10 µm GW788388”. It should µM…please be accurate through the whole Ms and proof read.**

**2. Line 215, 298, 968, 970 etc,, “western blotting” should be “Western” with capital “W”**

**4. Line 285, “Taqman Gene Ex Assay” should be Taqman Gene Expression Assay**

**8. Line 983, please specify which TGF (b1, 2, 3?)**

**10. Line 989 “TGF inhibitor GW788388” is it TGF-beta or TGF receptor inhibitor?**

**12. Line 996, “C-D: expression of the genes encoding PAR1 (C) and endothelin-1 (D) after the treatments in (A).” Please specify treatment with what.**

We apologize for the imprecisions, which have been corrected.

**7. Line 333, “1/2^DCt 0.03 for PAR1, 0.0001 for PAR2 and 0.002 for PAR3”. This information should not be used in the Ms unless shown in the figures (which is currently not included).**

**11. Line 994, “P for trend <0.0001 in one-way ANOVA, not presented in the figure for simplicity and clarity sake”. Please do not report in the figure legend what is not presented/reported in the figure. Please remove this sentence.**

**13. Line 1107, “** is P <0.01 and ***”. Please remove “is”.**

The suggested changes have been made.

**3. Line 257, how was picro-sirius red quantified?**

The information has been added (see also above): “*Collagen synthesis was also analyzed by Picro-Sirius red staining of Fib cultured in 8-well chamber slides; red signal intensity was quantified in images taken with a conventional light microscope.*”

**5. Line 288, “comparative Ct method”- please be specific and state which exactly method was used (DDct, DCt….etc)**

We used the DDCt method, it is now stated in the text.

**6. Line 317-325, how was the non-normally distributed data expressed (mean±SEM cannot be used for non-normally distributed values)? This is not clear.**

All data are presented as mean ± SEM, as it is commonly done in basic science papers.

**9. Line 967, 1039 (and through the whole text) “Magnification of pictures is 400x and bars” – was it 400x or 40x?**

Magnifications in the figure legends are correct.
